# Oncological and functional outcomes of transoral laser surgery for hypopharyngeal carcinoma

**DOI:** 10.1007/s00405-022-07622-1

**Published:** 2022-09-03

**Authors:** Rodrigo Casanueva, Fernando López, Patricia García-Cabo, César Álvarez-Marcos, José Luis Llorente, Juan Pablo Rodrigo

**Affiliations:** 1 Department of Otolaryngology, Hospital Universitario Central de Asturias, University of Oviedo, Avenida de Roma, s/n, 33011 Oviedo, Spain; 2 Instituto de Investigación Sanitaria del Principado de Asturias, Oviedo, Spain; 3 Centro de Investigación Biomédica en Red de Cáncer (CIBERONC), ISCIII, Madrid, Spain; 4 Instituto Universitario de Oncología del Principado de Asturias, University of Oviedo, Oviedo, Spain

**Keywords:** Hypopharyngeal cancer, Transoral, Laser, Head and neck cancer, Surgery

## Abstract

**Background:**

Surgical resection or radiotherapy (RT) are standard approaches for early-staged hypopharyngeal squamous cell carcinoma (HPSCC). Transoral laser microsurgery (TOLMS) seems to provide good oncological and functional results with few local complications. The aim of our study was to analyze the outcomes of TOLMS, with or without neck dissection or RT, in the treatment of HPSCC in a tertiary referral center.

**Methods:**

A retrospective study was conducted in patients with early T-category (T1–T2) HPSCC treated by TOLMS.

**Results:**

A total of 34 patients were included in the study. The series includes 17 (50%) T1 and 17 (50%) T2 classified tumors. The 5-year overall survival and disease-specific survival rates were 51% and 66%, respectively, with a 5-year local control rate of 92%. All patients reassumed oral diet and none of them had a tracheostomy at the end of the follow-up.

**Conclusions:**

TOLMS offers an effective treatment option in terms of oncologic control and function preservation in locally circumscribed HPSCC.

## Introduction

Hypopharyngeal squamous cell carcinoma (HPSCC) constitutes 3–7% of all head and neck cancers and, despite advancements in surgery and chemo-radiotherapy (CRT), its prognosis remains poor, with one of the highest associated morbidity and mortality of any head and neck squamous cell carcinomas [1–3].

Most patients with HPSCC (70–85%) present with advanced-stage disease (stages III and IV), as a result of the local extension of the tumor and the high incidence of lymph node involvement at the time of diagnosis [4]. 50–70% of patients show clinically positive cervical nodes, 5–10% have distant metastasis and 20% of patients are considered inoperable at diagnosis. Up to 60% of patients develop metastasis at some point throughout their treatment or follow-up [3, 5]. In addition, second primary tumors develop in 25% of cases [4]. Therefore, the 5-year overall survival (OS) rate is reported to be around 15–45% [3, 6].

Within this context, the aim of the treatment is to optimize survival and provide functional organ preservation, when feasible. Surgical resection or radiotherapy (RT) have become the standard approaches for early-staged tumors (stages I–II). On the other hand, the treatment for patients with locally advanced HPSCC (T3-T4) consists of a total laryngectomy with partial or total pharyngectomy or protocols based on CRT [1, 6].

RT (in early stages) or CRT (in advanced stages) does not jeopardize oncological outcomes and allows for satisfactory functional results, thus becoming the treatment of choice in most cases. However, avoiding surgery is not a guarantee of functional preservation, given the early and late side effects of RT/CRT [1]. Although it would seem evident that the quality of life would be superior, given that the structural framework of the larynx remains intact, radiation focused on the pharyngeal constrictors results in a significant risk of dysphagia and aspiration [7]. Consequently, a considerable number of organ-preserving surgical procedures have been developed and tested for select early-staged HPSCC, including CO_2_ transoral laser microsurgery (TOLMS) and transoral robotic surgery (TORS), as well as partial open procedures [3].

TOLMS as treatment for T1-T2 HPSCC has achieved excellent outcomes, such as those obtained with open surgical procedures and RT, with lower postoperative morbidity and optimal functional results [1, 3, 8, 9]. A recent meta-analysis has shown that there were no significant differences between TOLMS and RT for local control and for OS in early staged tumors, and suggests improvements in functional and oncological outcomes with TOLMS [2]. However, due to the scarcity of works on the subject, only 6 articles could be included in this meta-analysis, most of them with a small number of patients (only two of them had 50 patients or more), so the results should be interpreted with caution. In addition, the results of TOLMS in patients with locally advanced HPSCC are contradictory, the experience is still limited, and the role of this technique has not yet been defined.

In the attempt to provide information on the role of TOLMS in the treatment of HPSCC, the aim of our study was to evaluate the oncologic and functional outcomes of TOLMS for HPSCC at our Department in a tertiary referral center, and to determine the influence of the clinic-pathologic factors in the prognosis of these patients.

## Patients and methods

We reviewed the clinical records of the patients with HPSCC treated with TOLMS at our Department from 2000 to 2018. Written informed consent was obtained from each patient. Data were extracted from the original medical records, and they were retrospectively reviewed and analyzed anonymously, without any further inquiry or patient intervention, following institutional review board guidelines.

The variables included were age, sex, tumor location, tobacco and alcohol consumption, familiar and personal history of cancer, stage, histological grade, post-operative complications, and the incidence of recurrence and second primary malignancies. Tumors were classified according the TNM classification of the International Union Against Cancer (8th Edition, 2017). In terms of the magnitude of consumption, among the smokers the cumulative doses were calculated according to the pack-year index and among the drinkers according to the grams of ethanol consumed per day. The minimum follow-up of the patients included in the study was 24 months.

The surgical procedures were performed under general anesthesia after orotracheal intubation, with Sharplan 30C CO_2_ LASER System and AcuPulse CO_2_ LASER system. Endoscopic resection was performed using en bloc or piecemeal techniques according to several variables, including size and location of the tumor, as well as the tumor exposure. All the patients underwent a transoral laser CO_2_ microsurgery with curative intent, following Steiner´s recommendations [10]. The extension of the resection was tailored according to the emplacement and size of the tumor. An appropriate resection margin could be maintained of at least 5 mm [11].

After being assessed in a multi-disciplinary committee, TOLMS was indicated for all those patients with T1-T2 hypopharyngeal tumors once adequate exposure of the entire lesion was confirmed by microlaryngoscopy. Adequate exposure implies not only a complete view of the lesion, but also sufficient space to allow maneuvering the surgical instruments. In cases where complete resection of the tumor was deemed not possible transorally, a non-surgical treatment was chosen.

All patients were assessed by expert anesthesiologists to ensure a preoperative morbidity status good enough to withstand the procedure (ASA 1 or 2).

Ipsilateral neck dissection was carried out in patients with lateralized not crossing the midline tumors and no clinical disease on both sides of the neck. The indications for performing a selective neck dissection (levels II–IV) were absence of identifiable metastatic neck nodes or radiological findings of positive nodes without extracapsular spread. The remaining cases received a modified radical neck dissection.

During the neck dissection, the ipsilateral superior thyroid artery was prophylactically ligated to reduce the risk of massive hemorrhage. When a neck dissection was not included in the treatment plan, no additional measures were taken to reduce the risk of bleeding.

Patients with affected surgical margins, more than 2 positive nodes or extra-nodal extension in the pathological examination, received postoperative intensity-modulated RT within 3 months after surgery, following our hospital´s treatment protocols, which are based on the recommendations of the NCCN guidelines in use at the time. For tumors amenable to larynx-preserving surgery, these guidelines recommend either definitive RT or partial laryngopharyngectomy associated with ipsilateral or bilateral neck dissection, the latter being our treatment of choice. In case of adverse postoperative features, such as positive margins and/or extranodal extension, the NCCN guidelines contemplates RT as an acceptable adjuvant treatment.

The follow-up consisted of standard fiberoptic laryngoscopy every 3–6 months. A CT including neck and thorax was performed annually on all patients for the first 5 years of follow-up.

The endpoints assessed were OS, disease-specific survival (DSS) and the local control rate (LCR). ﻿For DSS, only death from pharyngeal cancer was seen as an event. Survival curves were drawn up according to the Kaplan–Meier product limit estimate. Differences between survival times were analyzed by the log-rank method. In evaluating recurrence, events were defined as local and regional recurrences, distant metastasis and combinations of them.

Functional outcomes were assessed based on the need for permanent tracheostomy or enteral feeding tube.

All statistical analyses were performed using IBM SPSS statistics software version 22 for MacOS. *p* < 0.05 was considered statistically significant for all comparisons.

## Results

The characteristics of the patients and their tumors are summarized in Table 1. A total of 34 patients were included in the study, 31 men (91%) and 3 women (9%). The mean age at diagnosis was 59.6 years (range 47–80 years). 26 patients were smokers (76%) and 25 had history of alcohol consumption (73%).

**Table 1:** Characteristics of the patient population and their tumors

Variable	No. cases (%)
Mean age (range)	59.6 (33–47) years
Alcohol consumption	
Unknown	9 (27)
< 50 g/day	3 (9)
50–100 g/day	11 (33)
> 100 g/day	10 (30)
Tobacco consumption	
Unknown	7 (21)
Never	1 (3)
< 10 pack-year	1 (3)
10–40 pack-year	11 (33)
> 40 pack-year	13 (39)
Sex	
Male	31 (91)
Female	3 (9)
Location	
Piriform sinus	31 (91)
Post-cricoid area	1 (3)
Posterior wall	2 (6)
pT classification	
T1	17 (50)
T2	17 (50)
pN classification	
N0	12 (35)
N1	4 (12)
N2	13 (38)
N3	5 (15)
Disease stage	
I	6 (18)
II	6 (18)
III	4 (12)
IVA	18 (53)
Histological grade	
Well-differentiated	12 (36)
Moderately differentiated	7 (21)
Poorly differentiated	14 (42)

Most tumors were localized in the piriform sinus (91%), followed by the postcricoid area (6%) and the posterior wall (3%). Primary tumors were equally distributed in T1 and T2 classifications. However, given the high proportion of patients with nodal metastases, 65% of patients presented an advanced tumor stage (III and IVA). Most tumors were poorly differentiated carcinomas (42%).

Surgical margins were microscopically involved in 4 out of 34 cases (12%). All cases with involved margins were T2 tumors.

31 patients (91%) underwent a neck dissection, which was performed simultaneously in 26 cases (84%) and staged in 5 (16%). A unilateral neck dissection was carried out in 23 patients (74%) and 8 (26%) underwent bilateral neck dissection. Except in 7 cases, where type II modified radical neck dissection was performed, a selective neck dissection (levels II–IV) was done. From the 31 patients that received a neck dissection, 19 (61%) presented pathological neck metastasis. 3 patients did not undergo neck dissection. All patients had a clinically negative neck and had a staged elective neck dissection planned, but declined it after surgery for the primary tumor.

Excluding two cases that died in the postoperative period, all patients with pathological neck metastasis (59%) received adjuvant RT (in one patient with concomitant chemotherapy).

Patients had a median postoperative hospital stay of 14 days (range 5–41 days). The major complications were: bleeding in 4 patients (11.7%), respiratory infection in 1 patient (3%) and airway obstruction requiring emergency tracheostomy in 2 cases (6%).

2 patients (6%) died in the postoperative period from massive hemorrhage. No differences were identified in either clinical or management aspects in these two patients with respect to the rest of the sample. Both patients were male, aged 59 and 73 years. One of them had a T1 tumor and did not receive a neck dissection, and the other had a T2 tumor and received a simultaneous neck dissection.

Among minor complications, 1 patient presented a chylous fistula, resolved with conservative treatment.

### Oncologic results

Excluding the two patients who died in the postoperative period, the mean follow-up time was 46 months (range 24–120 months). ﻿At the time of the last follow-up visit, 15 patients (47%) were still alive and free of disease, 8 (25%) had died due to intercurrent disease without evidence of recurrence and 9 (28%) had died with cancer.

Recurrent disease (including loco-regional recurrence and distant metastasis) developed in 10 patients (31%): 1 patient (3%) developed local recurrence and 5 of them (16%) had a regional recurrence. One patient (3%) developed both local and regional recurrences. Distant metastases were found in 3 patients (9%), all of them located in the lungs. Nine patients (28%) developed a secondary primary tumor: 5 (55%) of them located in the upper aerodigestive tract area (2 in the oral cavity, 2 in the larynx and 1 in other emplacement of the hypopharynx), while 4 of them (45%) were in the lungs. The patient with local recurrence was treated with total laryngectomy and adjuvant RT and subsequently developed lung metastases. The 3 patients with regional recurrence that did not receive postoperative RT were treated with salvage radical neck dissection and adjuvant RT; two of them had additional recurrences and received palliative treatment. The patient with both local and regional recurrence was considered unresectable and received palliative treatment with chemotherapy, as the patients with regional recurrences that received adjuvant RT and the patients with distant metastases.

No statistically significant differences were found between the presence of loco-regional recurrence and the T and N classifications, nor with the histological grade or the margin status. However, recurrences were higher in patients that did not receive adjuvant treatment, reaching near statistically significant differences (Table 2). In contrast, all the patients that developed distant metastasis had received adjuvant treatment.

**Table 2:** Relationship between pathological and treatment variables and loco-regional recurrences

Variable	Total cases	Recurrence (%)	*p*
pT classification			
T1	16	3 (19%)	0.67
T2	16	4 (25%)	
pN classification			
N0	11	3 (27%)	0.6
N1–3	21	4 (19%)	
Histological grade			
Well-differentiated	12	3 (25%)	0.8
Moderately differentiated	7	2 (28%)	
Poorly differentiated	13	5 (38%)	
Surgical margins			
Free	29	7 (24%)	0.34
Involved	3	0	
Adjuvant treatment			
No	13	5 (38%)	0.06
Yes	19	2 (10%)	

**Fig. 1:**
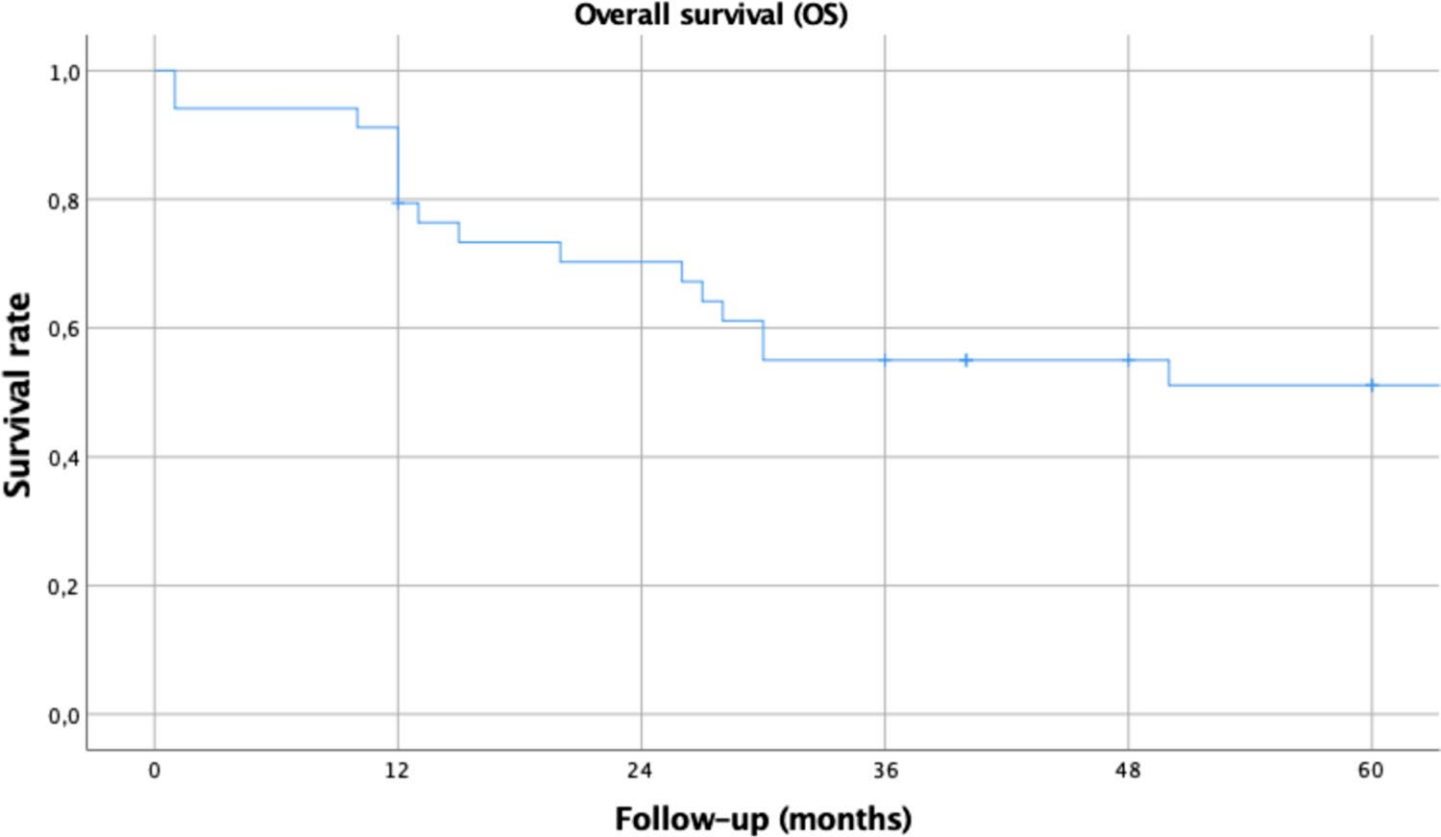
5-year Kaplan–Meier estimates for overall survival related for 34 patients with HPSCC

**Fig. 2:**
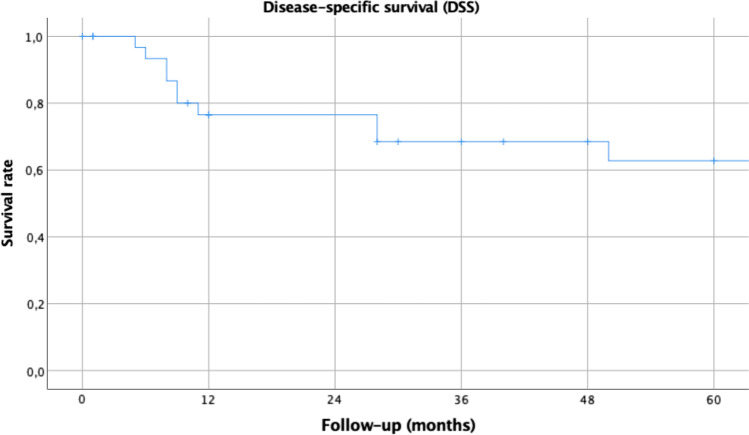
5-year Kaplan–Meier estimates for disease-specific survival related for 34 patients with HPSCC

The 5-year OS (Fig. 1) and DSS (Fig. 2) rates were 51% and 66%, respectively, with a 5-year LCR of 92%. No statistically significant differences were found in OS nor DSS regarding T classification, N classification, disease stage, margins status, histological grade or adjuvant treatment (Table 3).

**Table 3:** 5-year overall and disease-specific survival rates in relation to pathological factors and treatment

Variable	5-year OS^a^ (%)	*p*	5-year DSS^b^ (%)	*p*
pT classification				
pT1	58	0.67	78	0.42
pT2	44		54	
pN classification				
pN0	64	0.46	63	0.68
pN1-3	43		71	
Disease stage				
I–II	64	0.58	70	0.79
III	37		37	
IV	44		71	
Histological grade				
Well-differentiated	50	0.46	83	0.43
Moderately differentiated	43		71	
Poorly differentiated	36		64	
Margin status				
Free	48	0.35	62	0.26
Involved	75		100	
Adjuvant treatment				
No	57	0.77	65	0.9
Yes	47		67	

^a^Overall survival

^b^Disease specific survival

### Functional results

A nasogastric feeding tube was needed in 26 patients (76%) in the postoperative period. The remaining patients reassumed oral diet the day following surgery. The mean time to achieve oral feeding was 7.4 days (median 6 days) and the mean duration of nasogastric tube feeding was 7.2 days (median 5 days). Nasogastric tube could be removed in 25 patients and 1 patient (3%) required a temporary gastrostomy, that could be removed 6 months later. Two patients required a tracheostomy, that could be also removed, and therefore, none of the patients had a gastrostomy or tracheostomy at the end of the follow-up. Only one patient required a total laryngectomy (due to a local recurrence), so the 5-year laryngectomy-free survival rate was 97%.

## Discussion

Patients with primary HPSCC classified T1 or T2, without nodal or distant metastases may be treated with transoral approaches, open partial laryngectomy, or RT. Local surgical treatment is usually accompanied by neck dissection and adjuvant RT when indicated. The lack of prospective randomized trials makes it difficult to compare these approaches. In addition, the different stages, end points, observation periods, statistics and indication criteria of the different treatment modalities make it difficult to compare the efficacy of one treatment over another. In this study we assessed the oncologic and functional results of TOLMS for primary management of HPSCC.

Even though TOLMS offers a surgical option in these tumors, because all regions of the hypopharynx are accessible with this approach [10], studies in the literature are scarce. Our series consists of 34 patients, with T1 and T2 classified tumors and TOLMS has demonstrated to be an effective treatment in these cases, with a 5-year local control rate of 92%. However, as many patients have nodal metastasis, most patients (53%) have tumors in stage IV of the disease, resulting in a 5-year DSS of 66% and a 5-year OS of 51%. These results are comparable to those reported by other authors. Survival and local control rates of the most relevant published studies are summarized in Table 4 [11–21]. 5-year OS ranges from 37 to 77%, 5-year DSS ranges from 58 to 88% and LCR ranges from 71 to 87%.

**Table 4:** Overview of main studies evaluating oncologic results in HPSCC treated by TOLMS

Study (year)	n	T category	5-year OS^a^	5-year DSS^b^	5-year LCR^c^
Present study (2022)	34	T1–2	51%	66%	92%
Rudert and Höft [14]	29	T1–4	48%	58%	72%
Steiner et al. (2001) [32]	129	T1–4	71%	–	–
Vilaseca et al. (2004) [17]	28	T1–4	43%	59%	87%
Martin et al. (2008) [33]	172	T1–4	–	60%	71%
Karatzanis et al. (2010) [19]	119	T1–2	–	73%	85%
López et al. (2011) [18]	16	T1–3	44%	62%	–
Kuo et al. (2013) [17]	25	T1–3	67%	76%	–
Canis et al. (2013) [12]	25	T1–4	37%	88%	60%
Weiss et al. (2017) [11]	211	T1–4	55%	74%	76%
Breda et al. (2017) [16]	37	T1–4	50%	72%	74%
Hung et al. (2018) [13]	46	T1–4	77%	83%	–

^a^Overall survival

^b^Disease specific survival

^c^Local control rate

We obtained an excellent LCR of 92% at 5 years, superior to the rates described in the other studies (Table 4) and the 78% pooled local control rate described in the meta-analysis of Lane [2]. Our better LCR could be explained, because we only included tumors classified T1 and T2, whereas the other reports about TOLMS (except the one from Karatzanis et al. [19]) also included a variable proportion of tumors classified T3 and T4 (6.9–62%). This could also explain that our series is also one with the lowest rates of postoperative RT/CRT: 59% of patients, whereas all the remaining series of TOLMS for HPSCC report adjuvant treatment in more than 70% of patients, except for the study of Vilaseca et al. [17], with postoperative RT in 43% of patients. This highlights the importance of an adequate selection of patients to obtain the best oncologic results with the lowest morbidity. The better LCR in the studies that only included T1-T2 tumors suggest that TOLMS is best indicated in this subset of patients. However, the results of the studies that also included locally advanced tumors compare well with those obtained with other treatments. A recent multicenter trial found LCR of 82% and 63% for traditional pharyngolaryngectomy and CRT, respectively [20]. These data suggest that there is no inferiority for local control for selected patients treated with TOLMS.

Although the LCR obtained with TOLMS is adequate, OS and DSS are compromised due to the high incidence of nodal metastasis (which results in more than 2/3 of patients being in advanced stages) and the frequent development of distant metastasis. Then, the described 5-year OS and DSS are between 37–77% and 58–88%, respectively (Table 4). These oncologic results appear comparable with that of open approaches, with a 5-year OS rate of around 60% to 75% in stage I and II disease and 40% to 50% in stage III and IV disease. Moreover, Kuo et al. [15] compared 2 cohorts with HPSCC treated by TOLMS or open partial laryngopharyngectomy in a single institution study. The 3-year OS and DSS rates were comparable between TOLMS and open surgery (79% vs 64%, *p* = 0.151, and 83% vs 71%, *p* = 0.320, respectively). However, the TOLMS had higher laryngeal preservation rates (92% vs 71%, *p* = 0.048) and better functional outcomes. Although these results are based mostly on small case series, they suggest that TOLMS offers equal, if not better, oncological results than open approaches in the treatment of HPSCC. In addition, among the advantages of TOLMS when compared with open approaches, the main one could be the early recovery of pharyngolaryngeal functions. In most patients, tracheotomy is not necessary and healthy ﻿tissue may be maximally preserved to reduce morbidity, as reported by Suarez et al. [9]. Moreover, for the healing process no reconstruction is needed, thus reducing considerably the duration and costs of the procedure.

Primary RT/CRT might also be a treatment option for HPSCC that would be surgically resectable. Modern techniques and protocols follow the goal of best tumor control, such as maximal sparing of healthy tissue to diminish toxicity. In early-stage disease, primary RT achieves good oncologic outcome and is an alternative treatment strategy. Nakamura et al. [21] reported excellent oncological results in early HPSCC treated with RT. They found a 5-year DSS rate of 95.8% for patients with T1 disease and 70.1% for patients with T2 disease (*p* = 0.02). Rabbani et al. [22] reported 85% LCR for T1-T2 tumors treated by definitive RT, although the DSS and OS were lower (61% and 35%) as in cases treated with surgery. In stage I–II HPSCC treated with RT/CRT, Sato et al. [24] reported a 5-year OS rates, DSS rates and LCR rates of 58%, 75% and 56%, respectively, and Mendenhall et al. [24] reported a 5-year LCR of 85%, DSS of 62% and OS of 38% in 135 T1-T2 HPSCC treated with RT.

Keeping in mind that early tumors usually have better prognosis, the goals of treatment should be not only to cure, but also to maintain adequate swallowing and voice function. According to Alvarez-Marcos et al. results, patients with tumors of the larynx and hypopharynx treated with CRT have frequent asymptomatic swallowing disorders that alter their quality of life [7]. It is, therefore, important to look for the treatment that achieves the best oncological and functional outcomes. In this regard, only one of our patients required a temporary gastrostomy and two a tracheostomy, but in all of them they could be removed. In contrast, in the review from Lane et al. [2], gastrostomy ﻿rates ranged from 2.2 to 17%, with a median of 95% patients achieving oral intake, while tracheostomy rates ranged from 0 to 6% (median 3%). These results compare well with functional reports of other treatments in HPSCC. A comparative study between initial surgery and RT/CRT found a feeding tube dependance of 11.1% and 4% in early stage (I–II) patients treated with surgery and RT, respectively, whereas in advanced stage (III–IV) patients, these rates were of 7% for surgical treatment and 13% for CRT [25]. Other study that included patients with HPSCC classified T1-T2 treated with RT/CRT reported even higher rates of tracheostomy (19%) and gastrostomy (41%) [27]. In line with the best functional results reported with TOLMS, Tsung-Lun et al. [27] found that ﻿TOLMS may provide comparable, if not better, quality of life related to physical effects for patients relative to open surgery and RT/CRT.

The organ preservation rate in our series was 97%, with only one patient requiring a total laryngectomy due to tumor recurrence. This high laryngeal preservation rate was also described in the systematic review of Lane et al. which report laryngeal preservation rates from 89 to 100% (median 97%) [2]. These results are better than those obtained with open partial surgery, as demonstrated in the comparative study of Kuo et al. [15] and other reports of open surgical approaches [28, 29], which describe laryngeal preservation rates around 70%.

These results, compare well with the laryngeal preservation rates obtained with RT/CRT, with reported rates between 56 and 87% [21, 22, 25, 26]. Moreover, and differentially, TOLMS offers ﻿the possibility of obtaining prognostic information from the surgical specimen. Precise data about tumor characteristics are of crucial importance. It may allow us to administer adjuvant treatment in a more rational manner, thus reducing the risk of overtreatment and the complications associated with that [30].

As observed in our series, adjuvant treatment can be avoided in a large proportion of patients (41%), which may explain the excellent functional results without compromising oncologic outcomes. The high incidence of second primary tumors in this kind of patients represents another reason to avoid unnecessary adjuvant treatments and preserve the option of RT/QRT for such scenarios.

However, although no significant differences were identified, a trend toward higher incidence of recurrence was observed in patients who did not receive adjuvant treatment (Table 2). Further studies must be conducted to clarify the role of adjuvant treatment in these tumors, overcoming the inherent limitation of our study, which is the sample size.

The restriction to obtain wide resection margins is an inherent limitation of this surgical technique compared to open approaches and TORS [31]. We were able to develop a macroscopic complete excision in all cases, achieving a R0 status in 88% of them. Karatzanis et al. [19] reported achieving negative surgical margins in 84% of their sample, finding that survival rates were significantly better for this R0 cases. No statistically significant differences were found in our sample in this regard, which could be attributed to the administration of postoperative RT in these cases.

One advantage of TOLMS is the low complication rate. In our study, the most common complications were postoperative bleeding that occurred in 11.7% of patients. This is in line with the results of another study investigating TOLMS for HPSCC [17]. Although in most cases can be managed by transoral electrocautery or clipping, in our series 2 patients died because of a massive hemorrhage. It should be noted that these two cases occurred in the first years of performing this surgical technique, with no further cases of massive hemorrhage in the last 15 years, which may reflect the learning curve required when implementing a new technique. Significant arterial bleeding in these patients can be life-threatening, because tracheostomy is usually not performed. Therefore, extreme care must be taken to ensure that hemostasis is as thorough as possible. No additional patient- or intervention-associated factors could be identified to help explain these diseases.

## Conclusions

TOLMS offers an effective treatment option in terms of oncologic control and function preservation for locally circumscribed (T1-2) HPSCC, with similar oncological results to open approaches or CRT and improved functional outcomes.

## Data Availability

Authors declare that all data and materials are available for review.

## Code availability

Statistical analyses were performed using IBM SPSS statistics software version 22 for MacOS.
